# Mobile-Cloud Assisted Video Summarization Framework for Efficient Management of Remote Sensing Data Generated by Wireless Capsule Sensors

**DOI:** 10.3390/s140917112

**Published:** 2014-09-15

**Authors:** Irfan Mehmood, Muhammad Sajjad, Sung Wook Baik

**Affiliations:** College of Electronics and Information Engineering, Sejong University, Seoul 143-747, Korea; E-Mails: irfanmehmood@sju.ac.kr (I.M.); sajjad@sju.ac.kr (M.S.)

**Keywords:** wireless capsule sensor, video summarization, mobile-cloud computing, energy saving, remote monitoring, implantable sensors

## Abstract

Wireless capsule endoscopy (WCE) has great advantages over traditional endoscopy because it is portable and easy to use, especially in remote monitoring health-services. However, during the WCE process, the large amount of captured video data demands a significant deal of computation to analyze and retrieve informative video frames. In order to facilitate efficient WCE data collection and browsing task, we present a resource- and bandwidth-aware WCE video summarization framework that extracts the representative keyframes of the WCE video contents by removing redundant and non-informative frames. For redundancy elimination, we use Jeffrey-divergence between color histograms and inter-frame Boolean series-based correlation of color channels. To remove non-informative frames, multi-fractal texture features are extracted to assist the classification using an ensemble-based classifier. Owing to the limited WCE resources, it is impossible for the WCE system to perform computationally intensive video summarization tasks. To resolve computational challenges, mobile-cloud architecture is incorporated, which provides resizable computing capacities by adaptively offloading video summarization tasks between the client and the cloud server. The qualitative and quantitative results are encouraging and show that the proposed framework saves information transmission cost and bandwidth, as well as the valuable time of data analysts in browsing remote sensing data.

## 1. Introduction

Telemonitoring facilitates the delivery of healthcare services by providing the transmission of diagnostic information and consultation opportunities to/from remote patients. In telemonitoring, the most widely used sensors are spirometers, blood pressure, and heart monitors. These sensors are attached to the patient by wires, resulting in the subject becoming bed-bound. With the advent of wireless body sensors these requirements have been overcome, thereby enabling the patient to follow his daily routine during diagnosis procedures [1,2]. Wireless capsule endoscopy (WCE) [3] is an example of implantable wireless body sensors. WCE allows the diagnosis of remote patients, which increases their access to healthcare and decreases the healthcare delivery costs. WCE is a swallowable technology designed primarily to provide diagnostic imaging of the whole digestive tract. In WCE, the patient swallows a pill-sized capsule that includes a tiny camera, light source, RF transmitter, and batteries. The capsule films the entire gastrointestinal (GI) tract as it propels through the tract by normal peristalsis. The captured visual frames are transmitted by a tiny wireless sensor device to a portable wireless receiver located outside the human body. Current wireless capsule batteries have life spans of about eight hours [4], usually sufficient time for a capsule to image the entire GI. Although most wireless capsules are naturally expelled within 72 h of ingestion, only the first eight hours are significant for capturing the visual frames of the gastrointestinal. Therefore, a normal WCE diagnostic procedure lasts approximately eight hours. This produces some 50,000 image frames on an average, with a ratio of two frames per second. Visualization of the GI tract enables physicians to detect the diseases that are in early stages of development. In addition, the data collected when the patients are monitored remotely using body sensors is important for doctors to tackle any abnormal findings in a timely manner and to undertake appropriate measures.

WCE videos are large files containing redundant data, of which only a limited amount is useful for diagnosis. The camera of the wireless capsule captures mucosal images at different scales and orientations that results in the production of highly redundant data. Non-informative frames are created when the camera is exposed to turbid fluids and food substances. Thus, video summarization becomes essential in WCE because the large unrefined data becomes a bottleneck in terms of both storage and efficient browsing. In this context, video summarization is the most feasible solution that can save transmission costs and time for doctors in browsing a patient's information. The implementation of high-level signal processing solutions such as summarization on wireless WCE are not feasible because it has limited memory, energy, and computation capabilities. Moreover, the transmission of extensive video data prior to processing is unrealistic because it consumes high transmission energy. WCE videos can be analyzed on local servers such as smartphones. These servers can play a vital role as a WCE-coordinator and perform low-level computer vision tasks. Advancements in the hardware and operating systems of smartphones have made smartphones a superior development platform. These personal servers have an advantage of portability that allows patients with wireless sensors to engage in outdoor activities, thereby, rendering this as an ideal option for remote monitoring solutions. Recently, we have presented a visual attention-based WCE video summarization scheme [5]. This scheme is suitable for smartphones because it uses integral-image concept for efficient computation of visual saliency. However, the limited on-board computing, energy supply, and storage capabilities hinders the ability of smartphones to support long-duration remote monitoring applications. Mobile-cloud computing has emerged to overcome these limitations. Mobile-cloud computing can generate video summaries to deploy cost-effective pervasive healthcare systems.

In this paper, we present a mobile-cloud-assisted tele-endoscopic system (MCATS). The smartphone not only makes decisions on the offloading but also serves as a gateway for WCE to access cloud services via the Internet. During the offloading summarization tasks, the proposed system considers smartphone parameters such as bandwidth, battery, and user preferences. This provides resources-conscious and bandwidth-aware WCE monitoring. This facilitates the ubiquitous access of the semantically relevant data to authorized medical communities, allows searching for personalized trends and group patterns, offering insights into disease evolution and the rehabilitation process. In the rest of this paper, the terms “mobile devices” and “smartphones” are used interchangeably. The main contributions of the proposed work are:

- Presents a resource-conscious and bandwidth-aware framework to adaptively perform summarization of the video generated by wireless capsule sensors in reasonable time.
- Reduces transmission cost by summarizing data to avoid transmitting significant amounts of redundant and non-informative WCE videos.
- Allows patients to specify smartphone resource-specific summarization levels to perform processing on WCE data.
- Reduces rural practice isolation by providing ubiquitous access to the right information to colleagues, physicians, and specialists.

The remainder of this paper is organized as follows: Section 2 reviews related work. Section 3 describes the proposed framework. The experiments and results are discussed in Section 4. Finally, Section 5 concludes the paper.

## 2. Related Work

In this section, biosensors for health applications, video summarization techniques for biosensor data and mobile-cloud computing in health applications are described. Biosensors for health applications present a brief survey on the research and development done so far on biosensor systems for health-monitoring. The gradual advancement of a video summarization techniques have been presented to explore its role in medical field, because video summarization plays a vital role in the management and indexing of medical videos. In mobile-cloud computing we have presented state-of-the-art mobile-cloud based health monitoring schemes.

### 2.1. Biosensors for Health Applications

Progress in sensor technologies has initiated production of numerous new devices, revolutionizing the healthcare field [6]. These biosensors combined with wireless body area networks can remotely monitor patients at home or in the hospital. Tura *et al.* [7] developed a medical wearable sensor to measure heart rate and blood oxygen saturation through a pulse oximeter. This system focused on children with learning disabilities. It had a three-level architecture that stores data in a multimedia card. This data is transmitted at regular intervals to a personal server (home PC) via a Bluetooth wireless link. Finally, the data is transmitted from the personal server to a medical center via the Internet. Renard [8] has used implantable glucose sensors to monitor glucose levels in diabetic patients. For long term health monitoring, a distributed mobile application has been developed in MIT and Cambridge Laboratories [9]. It provides health monitoring with real time data processing and context classification. For monitoring they used wearable sensor acquisition boards, IR tags, accelerometer board, and body-media sensor wear. Lin *et al.* [10] presented a real time wireless physiological monitoring system for nursing centers. Its major focus was to monitor status of older patients by measuring blood pressure, heart rate and temperature. In [11], an implantable stress/strain sensor and pressure biosensors were developed for biomedical applications. These sensors consisted of a soft magnetic material and a permanent magnet. The *Code Blue* project developed by Harvard University provides cost saving, flexible, and high ease of use health care services in remote sites [12]. It captures information with wearable wireless sensor nodes to recognize human activity. This platform offers multi-patient monitoring environments based on ZigBee and Telos motes, including oximeter biosensors for motion activity. Iddan *et al.* [13] developed a novel wireless capsule sensor, which for the first time allowed painless endoscopic imaging of the whole small bowel. Toennies *et al.* [14] presented a detailed survey on methodologies that deal with wireless capsule sensor for healthcare. These biosensor-based health monitoring applications fail to deal with the large amount of data captured by biosensors. Since, biosensors have limited computational and communication resources, it is important to develop automatic image analysis algorithms to assist identification of the few diagnostically useful images among the huge amount of images retuned from the sensors. These methods must be efficient both in terms of power and time, while retaining the essential information. In this context, we aim to present an efficient mobile-cloud assisted WCE video analysis scheme that extracts non-redundant and informative images.

### 2.2. Video Summarization

Biosensor-based patient monitoring systems produce large amount of sensing data. Challenges that emerge from enormous amount of biosensors data are of ever increasing importance [15]. In health monitoring applications, the focus has recently been shifting from data acquisition to data analysis methods in order to provide more valuable information to the end users [16]. Considering the data analysis techniques for biosensors, most of them are related to clustering, classification, regression, and summarization [17–19]. Video summarization schemes provide ease in accessing the relevant content of medical diagnostic data [20,21]. Iakovidis *et al.* [22] proposed a video summarization approach for reduction of WCE reading time. This scheme is based on an unsupervised data reduction algorithm. In [23], a method of epitomized summarization of the WCE video for visual examination by gastroenterologist is presented. The epitome model can generate a condensed summary of the original video. To ensure the necessary visual quality of the generated epitome for clinical examination, they introduced the constraint for local context preservation. A WCE video summarization aiming reduction of the inspection time of gastroenterologists has been proposed in [24]. This scheme incorporates color histogram in Lab color space to represent each frame in a WCE video. Then, based on the color histogram, differences between two frames are computed, which leads to redundancy elimination. For a detailed review of the existing computer-vision based WCE video analysis schemes, the readers may refer to a review [25]. Due to limited resources, it is impossible for biosensors to perform computationally intensive data summarization tasks. To solve this problem, we have presented [5] a video summarization framework, pushing computational tasks to patients' smartphones for fast and effective computation. However, analysis of the sensor data with mobile-assisted health monitoring systems is challenging due to limited resources for data processing and communication. Researchers have incorporated mobile-cloud computing in wireless BANs to process the diagnostic videos efficiently [26–28]. Mobile-cloud computing provides scalable resources to perform computationally expensive tasks such as video summarization. This assists in resourcefully administering the remote monitoring and diagnosis procedures. The proposed framework presented in this article utilizes the capabilities of both video summarization and mobile-cloud technology for efficient processing and dissemination of medical data.

### 2.3. Mobile-Cloud Computing in Health Applications

In recent years, there has been a remarkable amount of work on the topic of mobile-cloud computing. It mainly focuses on the possibility to offload mobile phones tasks into the cloud server, extending system lifetime by reducing the computational burden of mobile devices. Gu *et al.* [29] have performed extensive trace-driven evaluations which showed that efficient offloading inference engines can effectively reduce resource constraints from mobile devices with far lower overhead than other common approaches. In [30], the authors presented an effective offloading service for resource constrained mobile devices. They considered a combination of multiple resources including CPU, memory, and bandwidth resource and aimed to reserve these mobile resources as much as possible. Miettinen and Nurminen [31] explained that energy efficiency is a primary consideration for mobile devices and argued that mobile-cloud computing architecture has the potential to save energy through offloading. Fortino *et al.* [32] proposed a framework that supports the development of cloud assisted body sensor applications. It is a multi-tier architecture that integrates body sensors data streams, middleware, and cloud computing. It enables large-scale sensor data processing and sharing among users in cloud and mobile devices. This work also presented a case study for the real-time monitoring and analysis of ECG data streams. In [33], the authors have given a detailed review of research work on mobile and cloud computing in the field of telemedicine. Based on the review of state-of-the-art techniques, the authors discussed the limitations of current technological development and suggested that cloud computing and mobile technology should be combined because mobile teleconsultation requires high speed data delivery and a big data center where patient data can be delivered, stored, retrieved, and managed securely. In [34], authors presented an energy-efficient method of adaptive resource discovery in mobile cloud computing. According to varying network environments, it adapts between centralized and flooding modes to save energy. Eli and Young [35] concluded that adaptive offloading can optimize future mobile application energy efficiency in the cloud. Therefore, for WCE video summarization we have presented a novel adaptive-offloading approach which considers user preferences, device specifications and the available network resources to optimally partition the application and data.

## 3. Framework of the Proposed System

**Figure 1.**
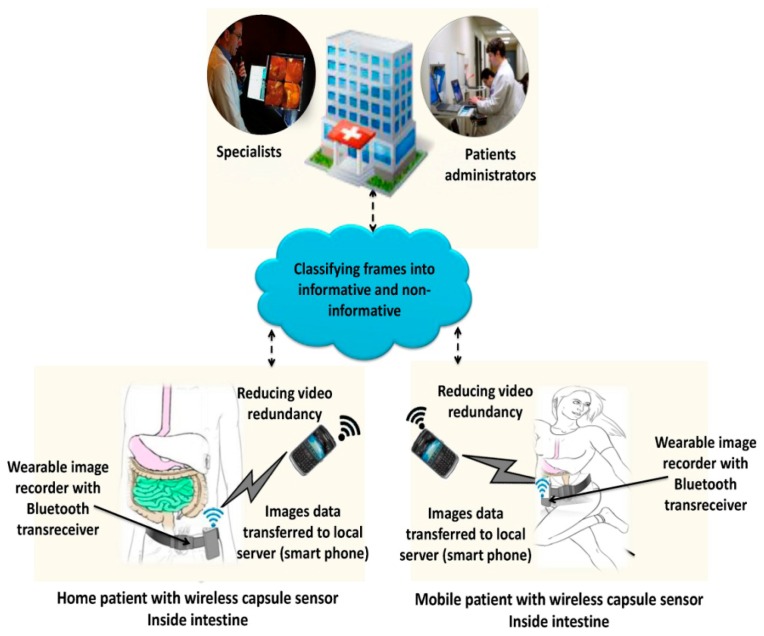
Conceptual view of the proposed summarization driven WCE monitoring framework.

WCE has emerged as a promising technology to monitor patients' GI tracts both locally and remotely. During the WCE process, significant amounts of video data are produced, however, only a limited amount of this data is useful for diagnosis. The sharing and analysis of this large amount of data is a challenging task. In order to facilitate efficient WCE data collection and browsing, we present a video summarization driven WCE framework that estimates the semantically relevant video frames. However, because of limited resources in terms of battery life and computational power, the implementation of video summarization on WCE is not feasible. Therefore, we incorporate mobile-cloud computing because it provides massive computation, storage, and software services in a scalable manner at low-cost. The proposed framework consists of: (1) a smartphone with a trusted communication gateway to collect the capsule endoscopy images; (2) endoscopic data summarization with adaptive-offloading from the smartphone to the cloud; (3) cloud computing, a trusted entity for computing and providing integrated storage and authorized access to patient's information ubiquitously. The overall view of the proposed system is shown in Figure 1.

Data collected from an implanted wireless capsule is received by smartphone via a portable image recorder unit (IRU). The IRU is usually fixed in a belt around the patient's waist. It facilitates the patient to perform their daily routine tasks simultaneously without carrying burdensome wired heavy devices. The built-in Wi-Fi facility and high battery life as compared to body sensors, provides ease of access to cloud services at low-cost and for a long duration [36]. The images received by the smartphone are summarized with adaptive-offloading between the client and cloud server. The summarized data may be logged into the cloud and registered using the patients ID for analysis. This enables doctors to regularly monitor the WCE procedure: in case of any abnormality, they can communicate with the patients. If needed, the patient can be instructed to visit a healthcare center.

### 3.1. Data Logger for WCE Videos

**Figure 2.**
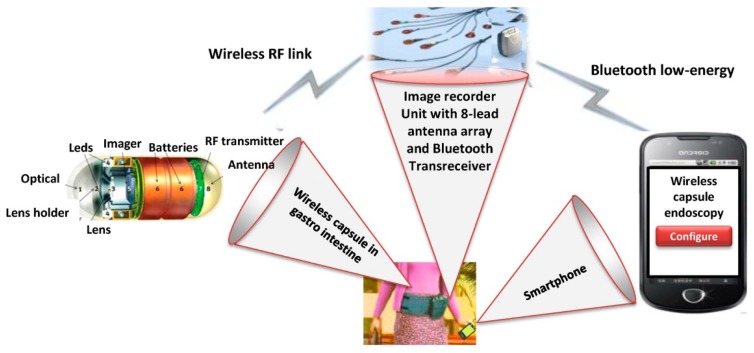
Smartphone data logger for wireless endoscopy.

WCE is a very useful technology that helps gastroenterologists to examine the human digestive tract for various abnormalities such as blood pressure, ulcers, and polyps. The wireless capsule can be swallowed easily without discomfort owing to its small size. The micro imager of the wireless capsule captures, compresses, and transmits images to the IRU using a radio frequency (RF) as shown in Figure 2. Patients undergoing WCE wear an antenna array consisting of various leads that are, connected by wires to the IRU. The antenna sensors are physical receptors that receive transmission data from the wireless capsule and transfer it to the IRU. Although there are different options made available by WCE manufacturing companies, 8-lead antenna array are the most widely used and are more effective [37–40]. The 8-lead antenna array is taped to the patient's anterior abdominal wall at eight specific points according to a standard designated pattern, as dictated by a template for lead placement [37]. The antenna array and IRU are usually worn under regular clothing. The processing and communication capabilities of the IRU are inadequate to manage large amount of data collected from the wireless capsule. Thus, there is a need to forward these captured visual frames to a smartphone. This is because smartphones provide local computational services and easy access to cloud services.

In order to transmit visual frames from the IRU to a smartphone, a low-cost and reliable transmission mechanism is required because of the limited resources of both the IRU and the smartphones. The most commonly used standards for wireless BAN communication includes: ZigBee, Ant, Ultra Wide Band (UWB), Bluetooth, and custom protocols [41]. ZigBee was initially developed for smart home applications. It is a low-cost, low power (60 mW) standard communication with a limited bandwidth. Because of its limited data rate (250 kbps), ZigBee is not feasible for transmission of multimedia contents such as capsule endoscopic images that require high data rates. Ant is a low-speed and low-power communication protocol. It is one of the emerging standards for health monitoring systems. Unfortunately, Ant has no support for any smartphone platforms. UWB has a high bandwidth with spatial localization of transmitters as compared to ZigBee; however, it is very complex in terms of usage and deployment, especially for the receivers on smartphone terminals. In the current scenario, Bluetooth is the most suitable option because of its high bandwidth and support on many smartphone platforms. In [42], a high bit-rate data logger and its connectivity through Bluetooth are presented. A Bluetooth transreceiver [43] is connected with an IRU microcontroller in WCE. This Bluetooth transreceiver is based on a Bluetooth low-energy protocol known as RN41 [44]. It is small, low power, simple to integrate Bluetooth radio for adding wireless capability to various bio-sensors. The RN41 is perfect for battery powered applications such as WCE and by default is ready to use in the serial port profile configuration. It has the functionality of low-power sleep and wake-up. Current smartphones (Apple [45], Android [46] and Microsoft [47]) have Bluetooth low-energy capability with support for Bluetooth 4.0 technology. This Bluetooth low-energy enables smartphones to connect with IRU's Bluetooth low-energy and receive endoscopy video frames with extremely limited battery consumption. The purpose of data exchange between IRU and the smartphone is to analyze data for mining useful information and sending this information to medical specialists.

### 3.2. Adaptive Offloading-Based Video Summarization

**Figure 3.**
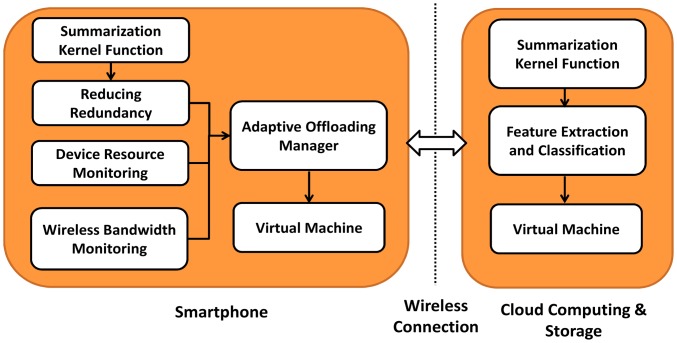
Architecture of the video summarization offloading service.

Current smartphones with limited resources are not practical for computationally expensive tasks and large data storage. Thus, to extract WCE video summaries, the computational intensive tasks can be offloaded to a cloud server. A virtual machine (VM) can support offloading by providing the capability to migrate partial or entire application from a smartphone to more powerful servers. This reduces the client (body sensors and smartphone) computational cost, thereby reducing energy consumption and increasing the lifetime of smartphone devices. However, the transmission of such large amounts of multimedia data results in high transmission cost. An efficient model is required to deal with this trade-off between computational and communication energy. Here, we present an adaptive-offloading to optimize the video summary execution time and energy consumption, as shown in Figure 3. This offloading architecture allows developers to select functions that need to be offloaded. These selected functions are known as kernel functions. The appropriate selection of kernel functions reduces the network traffic and computational load on smartphone devices. In our case, summarization of endoscopic images is considered as kernel function because of its computational complexity, which will be briefly explained in the Section 3.2.5. The details of each module in Figure 3 are given in subsequent sections.

#### 3.2.1. Mobile Device Resource Monitoring

For adaptive-offloading, it is necessary to analyze the usage of the smartphone resources. Significant changes in the device resources, triggers the adaptive-offloading manager. This module monitors the utilization of the mobile resources such as CPU and battery consumption.

#### 3.2.2. Wireless Bandwidth Monitoring

In remote monitoring applications, mobile devices transmit data over frequently changing bandwidths. However, the fluctuations in bandwidth create difficulties in continuous and robust monitoring. In low-bandwidth scenarios, majority of the computational tasks have to be performed locally; this affects the overall lifetime of the mobile and body sensors. Furthermore, bandwidth is considered a major factor in saving the energy of the overall system [48]. Therefore, we consider bandwidth as a parameter in our adaptive-offloading process to optimally partition the application and data for offloading. Wireless bandwidth monitoring module estimates the available bandwidth and the bandwidth required for collaborative applications. The bandwidth required for remote offloading depends on the total size of data and application code migrated between mobile and cloud servers. The offloading manager triggers the appropriate partitioning in accordance with the fluctuations in the available bandwidth.

#### 3.2.3. Adaptive-Offloading Manager

The offloading manager monitors the execution of kernel functions and analyzes periodically updated information such as the available bandwidth and device resources. Based on this analysis, the offloading manager decides the level of kernel function to be offloaded for remote execution. Users can control their smartphone resources consumption by monitoring the available resources and resources occupied by the application. The adaptive-offloading manager considers user preferences, device specifications and the available network resources. This approach meets the requirements of adaptability, configurability, and stability for optimal offloading.

#### 3.2.4. Remote Execution Manager

The remote execution manager is responsible for installing and maintaining applications on behalf of mobile devices. Under the supervision of a cloud manager, it can launch applications on different cloud nodes.

#### 3.2.5. Summarization Kernel Function

**Figure 4.**
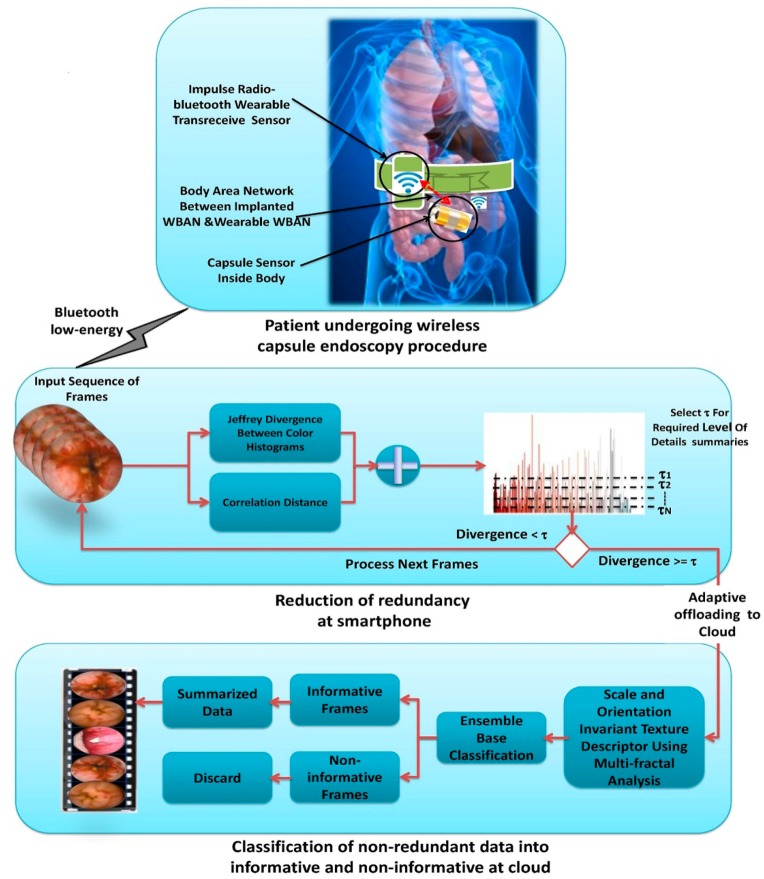
Illustration of the WCE data summarization framework.

The summarization kernel function is deployed at the mobile end as well as at the cloud end as shown in Figure 3. Taking into account the available bandwidth and resources of mobile devices, summarization kernel function divides summarization task into two steps: reducing redundancy and classification of non-redundant frames into informative and non-informative frames as shown in Figure 4. According to the well-known fact that the data transmission cost is significantly higher than the processing cost, transmission of complete WCE videos to cloud for summarization is not an intelligent option. Therefore, the redundancy removal task is performed at local server (smartphone) using Jeffrey-divergence (JD) and inter-frame correlation of color channels based on Boolean series that significantly reduces the size of the underlying data. The JD and Boolean series-based correlation are asymptotically light weight processes and consumes less energy that saves transmission energy in a substantial amount. However, the elimination of non-informative frames is a computationally expensive computer vision task (*i.e.*, feature extraction and classification), hence, it is offloaded to the cloud. On the basis of scale and orientation invariant texture features, ensemble-based classification is performed to separate informative and non-informative frames. Figure 4 shows the workflow of the proposed WCE summarization.

**Figure 5.**
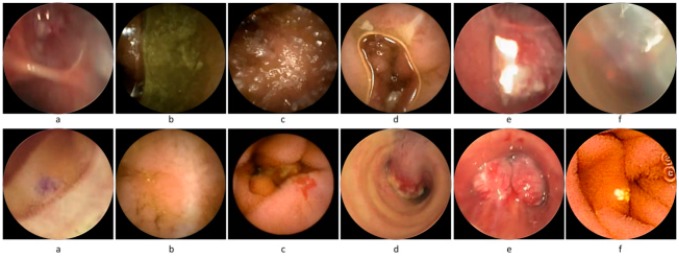
Sample frames from WCE videos: first row shows example of non-informative frames and second row shows informative frames.

The examination of WCE videos poses a tedious task for physicians because they have to perform a sequential scan over the video to extract informative frames for diagnosis. In WCE, numerous redundant and non-informative frames are generated as the capsule moves through various complex organs such as the stomach, small intestine, and large intestine. Redundancy in WCE videos occurs when images of the same mucosa are shot from different perspectives at different scales depending on the relative position of capsule imagery. The term non-informative frames may be defined as having invisible tissue. The non-informative frames are generated because of the wireless capsule exposure to different biological routes that are either too close or too far to focus on the mucosa colon. Coverage of the camera lens with foreign food substances also generates non-informative frames. The first row in Figure 5 shows various non-informative WCE frames found in different WCE videos.

These non-informative frames lack clear visualization of the underlying tissues because they are contaminated with various turbid secretions that include food, residual and faecal materials. The turbid secretions create a turbid layer over the GI tissues. As a result, the light for the wireless capsule is obstructed and the clear visualization of the GI tissues is hindered, as shown in first row of the Figure 5a–c. Recent studies have shown that non-informative frames are also generated due the appearance of bubble patterns either on capsule's camera or on tissues as shown in the first row of the Figure 5d–f. In the visual inspection of WCE videos, very little attention is paid to non-informative and redundant frames. Therefore, removal of all such non-informative frames is an important step in WCE diagnosis. Physicians are interested only in frames that visualize clearly the original tissue' characteristics of the GI tract as shown in bottom row of the Figure 5. These frames are considered to be informative frames because they provide a clear view for the diagnosis.

##### A. Reducing Redundancy

The proposed strategy for reducing redundancy is based on comparison of frames. In general, a single feature is usually not sufficient to estimate all the pictorial details of a frame and visual complexity of videos [49]. For an effective representation of pictorial contents of a frame, both the color and structural properties must be used. Therefore different features can be assorted to provide an effective representation of a frame. For this reason, two comparison measures are used: JD between color histograms and inter-frame correlation of color channels of adjacent frames. JD is an empirical measure of distribution similarity based on relative entropy. In [50], authors gave a detailed empirical comparison of nine dissimilarity measures that are based on distributions of color and texture features. They concluded that Jeffrey-divergence is more stable than other dissimilarity measures. Furthermore, various researchers [51–53] have proven that JD in contrast to other dissimilarity measuring algorithms is more stable with respect to noise and the size of the histogram bins. Scharcanski and Gaviao [54] proposed a diagnostic hysteroscopy video summarization framework that measures similarity between hysteroscopy video frames using JD. Their preliminary experimental results indicate that JD successfully removed redundant frames and played a major role in generating video summaries. Zheng and You [55] have also utilized JD for change detection in multi-temporal synthetic aperture radar images. Their experimental results proved that JD perform efficiently even in the existence of speckle noise and light variations. Therefore, JD is most feasible measure to eliminate the redundant frames generated during WCE procedure. To use color histogram in JD, the CIELAB or Lab [56] color system is selected because in this color space the differences among colors are more closely related to human perception than in spaces such as RGB, CIE 1931, *etc.* Color histograms are computed on each *L*, *a* and *b* channels. The histogram of each channel with intensity levels in the range [0, *L*−1] is a discrete function *h(r_k_)* = *n_k_*, where *r_k_* is the *k*th intensity values and *n_k_* is the number of pixels in the image channel with intensity *r_k_*. These color histograms fall in the category of color indexing according to the taxonomy proposed in [57]. After obtaining color histogram, a color quantization step is applied to reduce the size of the color histogram. The quantization of the color histogram is set to 16 bins for *L* and 4 bins for each of the *a* and *b* components. The *L*, *a* and *b* histograms are then normalized in the range of 0 to 1 by dividing each value by the maximum value in the respective component. The three histograms are then combined to get a histogram of size 24. Finally, the distance between frame *F_t_* and next frame *F_t_*_+_*_Δt_* is computed by using JD as follows:

(1) $$\begin{array}{r} D_{J}(H(F_{t}),H(F_{t+\Delta t}))=\overset{N}{\underset{i=1}{\text{∑}}}H^{i}(F_{t})*\log\left[\frac{2*H^{i}(F_{t})}{H^{i}(F_{t})+H^{i}(F_{t+\Delta t})}\right]\\ +H^{i}(F_{t+\Delta t})*\log\left[\frac{2*H^{i}(F_{t+\Delta t})}{H^{i}(F_{t})+H^{i}(F_{t+\Delta t})}\right] \end{array}$$

where *H^i^(F_t_)* and *H^i^(F_t_*_+_*_Δt_)* are the color histograms of the corresponding bin *i. F_t_* and *F_t_*_+_*_Δt_* are the successive WCE video frames with a time interval of Δt. *D_j_* provides a redundancy measure for video frames. For example, when wireless capsule gets stuck at a narrow spot in the digestive track, then static video segments are generated that are characterized by small inter-frame JD values *D_j_*. Based on the values of *D_j_* such static video frames are discarded. Correlation analysis is an important measure for the detection of duplicate frames in videos. However, the traditional methods are not suitable for real time applications [58–60]. In the current scenario, Boolean series-based correlation is used that works in real time with low computational complexity [61]. It efficiently computes the content changes in videos. For this purpose, first Boolean series of each channel *L*, *a*, and *b* are calculated as:

(2) $$\begin{array}{ll} B_{t}^{c}(\text{index}) & =\{\begin{array}{c} 1;F_{t}(\text{index},c)\geq F_{t-\Delta t}(\text{index},c)\\ 0;F_{t}(\text{index},c)<F_{t-\Delta t}(\text{index},c) \end{array};\text{where}c\in \{1,2,3\}\\ B_{t+\Delta t}^{c}(\text{index}) & =\{\begin{array}{c} 1;F_{t+\Delta t}(\text{index},c)\geq F_{t}(\text{index},c)\\ 0;F_{t+\Delta t}(\text{index},c)<F_{t}(\text{index},c) \end{array}\text{and}1\leq \text{index}\leq m*n \end{array}$$

The Boolean correlation *BC* coefficient of Boolean series is:

(3) $$BC(B_{t},B_{t+\Delta t})=\sum_{c=1}^{3}\left(1-\frac{\sum_{l=1}^{m*n}B_{t}^{c}(\text{index})⊕B_{t+\Delta t}^{c}(\text{index})}{m*n}\right)$$

here *m* and *n* is width and height of each frame respectively and *c* represents the color channels of CIELAB color space. ⊕ is a logical “Exclusive or” operation. It only returns true when both operands have different logical inputs *i.e.*, true/false and false/true. Here, in Equation (3), the same procedure is replicated.

We have incorporated the previous frames' dissimilarity measurements with the current frame using a weighted sum. Consider a frame *p*, its dissimilarity measurements: *δ* (the accumulative *D_J_* dissimilarity) and *ρ*(accumulative *BC*) with the previous *q* number of frames are calculated as:

(4) $$\delta (D_{J}^{p},D_{J}^{p-1},....,D_{J}^{p-q})=\frac{1}{q}\sum_{i=0}^{q}D_{J}^{p-i}*\frac{1}{i+1}$$

(5) $$\rho (BC^{p},BC^{p-1},....,BC^{p-q})=\frac{1}{q}\sum_{i=0}^{q}BC^{p-i}*\frac{1}{i+1}$$

This accumulated weighted sum produces dissimilarity measures that are robust to lighting changes and sudden camera motions. A linear fusion scheme is used to obtain the net distance *d(F_t_, F_t_*_+_*_Δt_)* between two consecutive frames as:

(6) $$d(F_{t},F_{t+\Delta t})=\delta (D_{J}^{t+\Delta t},D_{J}^{t+\Delta t-1},\ldots .,D_{J}^{t+\Delta t-q})+\rho (BC^{t+\Delta t},BC^{t+\Delta t-1},....,BC^{t+\Delta t-q})$$

Equation (6) illustrates that the net distance between two video frames is the linear combination of accumulative Jeffrey-divergence dissimilarity measure *δ* and accumulative Boolean series-based correlation measure *ρ*. This distance measured *d* is an effective parameter that helps to avoid extra computations by removing inliers. Inliers detection saves transmission costs by sending only representative frames to the cloud for further processing. Two frames *F_t_* and *F_t_*_+_*_Δt_*, are considered similar and included in the category of redundant frames if the dissimilarity *d* between them drops below a certain threshold *τ*, *i.e.*, *d (F_t_, F_t_*_+_*_Δt_)* ≤ *τ*. Small value of threshold is directly proportional to the number of frames that can be transmitted from smartphone to cloud and vice versa. As smartphone has variable resources that change from time to time (e.g., battery, bandwidth, *etc.*), therefore, adaptive thresholding is more suitable option compared to a fixed thresholding. This adaptive thresholding must be calculated by considering the smartphone resources such as bandwidth and battery due to following reasons:

- *Bandwidth (BW)*: The availability of high bandwidth usually increase the communication ability, therefore, to utilize the bandwidth effectively, the proposed system must measure the available bandwidth to adaptively set the flow of frames transmission. According to the proposed system, the small value of threshold will allow more frames to transmit from smartphone to cloud. Hence, the threshold must be formulated in a way that gives small value in case of high bandwidth and *vice versa*. To understand the wireless bandwidth monitoring process in detail, the readers are referred to *Android-er* [62].
- *Battery (B)*: Since, a significant amount of energy is required to transmit WCE video frames, thus, it is important to consider the available smartphone battery in measuring the value of threshold. High available battery can allow transmission of more frames by keeping the value of threshold small. On contrary, in case of low battery, transmission of frames can be reduced by keeping the value of threshold large. In the proposed framework, smartphone's battery level is monitored using Android SDK [63].

From above discussion, it can be concluded that smartphone's bandwidth and battery must be inversely proportional to threshold. Thresholding directly influences the usage of smartphone resources, that is, small τ will allow the proposed framework to utilize smartphone resources up to its maximum. To make the proposed system more interactive and controllable for user, user preference U is critical. It will be helpful for user to adjust the value of threshold according to the availability of resources. For example, in places such as home, office, *etc.*, where smartphone's battery can be easily charged and sufficient network link is available then user can set the value of threshold small for the transmission of more frames to cloud. Keeping in view the above considerations, a custom-defined equation for adaptive threshold τ has been formulated:

(7) $$\tau =e^{-\left(\sqrt{BW^{2}+B^{2}}\right)}*\text{U}$$

**Figure 6.**
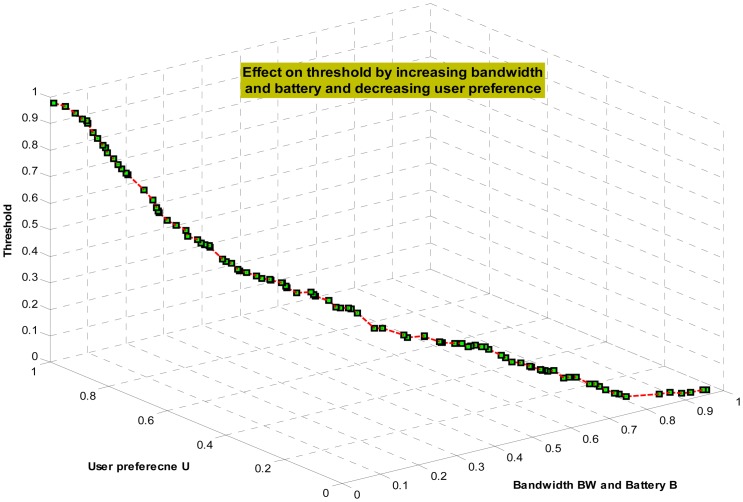
The combined variational impact of smartphone resources and user preference on threshold τ.

All the parameters *BW*, *B*, and *U* are normalized in the range (0, 1]. For example, a Samsung Galaxy S4 having full battery (2.6 mAh) is considered as 1 and similar is the case for other parameters. The graph given in the Figure 6 illustrates the combined variational impact of smartphone resources on threshold. From graph, one can see that threshold τ is inversely proportional to smartphone's resources. The increase of smartphone resources from minimum level 0 to maximum level 1, decreases the τ from a maximum value 1 to a minimum value 0. Thus, when we have enough smartphone's resources, then the computed value of the τ will be minimum which leads to elimination of only highly similar frames during redundancy removal step. This minimization of redundancy allows transmitting a large segment of frames to cloud. In contrary, when smartphone resources are limited then the resultant threshold τ value will be maximum that permits the discarding of frames even having little similarity. This results in the transmission of limited number of frames (highly non-redundant) to cloud. Thus, the proposed adaptive threshold reduces smartphone's computational and communication burden and only transmits limited number of frames to cloud. In addition, it enables adaptive offloading manger to efficiently utilize available resources.

##### B. Feature Extraction and Classification Based on Multi-Fractal Texture

Once the redundancy is removed, WCE video segments contain two types of frames: informative and non-informative. Frames showing original tissue's characteristics of the GI tract are considered informative, whereas the frames that are contaminated with food, faecal materials, or have an extremely far or close focus on tissues are considered non-informative. Therefore, it is important to detect and isolate these non-informative frames before generating the final summary.

Texture plays an important role in medical image analysis and understanding [64,65]. Texture features efficiently determine the granularity and repetitive patterns of different regions within a medical image. However, majority of the existing texture analysis methods work on the assumption that the underlying images are acquired from a fix viewpoint [66]. This limitation makes texture analysis schemes useless for medical images like WCE images, where, wireless capsule captures mucosa surface in GI tract from different perspectives and distances. Thus, a scale and orientation invariant texture features are highly significant for classifying WCE frames into informative and non-informative. In this context, multi-fractal texture features are the feasible option to classify the complex patterns of GI tissues as informative and non-informative. Multi-fractal offers rich description of the inherent structure and the texture of medical images at multiple orientations and scales [67]. Multi-fractal is the extension of fractal dimension and in statistics the fractal dimension of an image is a real number that describes the objects structures [68]. Takahashi *et al.* [69] presented multi-fractal-based classification scheme for early-stage detection of atherosclerosis disease. Similarly, Gonçalves *et al.* [70] proposed a classification scheme based on fractal dimension theory and deterministic partially self-avoiding walk. Numerous researchers have used multi-fractal theory for texture analysis and classification [67,71,72]. All these works validate the usefulness of applying the multi-fractal analysis to analyze medical images, especially to those captured under varying orientation, scale and translation conditions. Therefore, in order to classify WCE frame we extract multi-fractal-based texture features. Consider a set of points *C* in a 2D plane, for which the fractal dimension is:

(8) $$\dim(C)=\underset{r\to 0}{\lim}\frac{\log N(\sigma ,C)}{-\log(\sigma )}$$

where *N(σ,C)* is the smallest number of sets of diameter less than *σ* that spans *C*. Fractal dimension is the concept of analyzing an object's structural irregularities at different scales *σ*. It ignores the irregularities of size less than *σ* while studying an object at scale *σ*. For efficient categorization, it is necessary to define a categorization function specific to the task under consideration. Density function is one of the standard categorization functions that can be defined on various image intensity functions such as an edge filter. The local density function is:

(9) $$d(x)=\underset{r\to 0}{\lim}\frac{\mu (B(x,r))}{-\log(r)}$$

here *μ* is the measurement function applied to the Borel regular measure of a closed disk with centre *x* and radius *r*. The definition of *μ* is very important for correct classification. Consider *μ* as the sum of the Laplacian of the image inside the closed disc of radius *r* as:

(10) $$\mu (B(x,r))=\iint_{B(x,r)}\left|\nabla^{2}(G*F_{t})\right|dx$$

The Gaussian operator *G* is applied to reduce noise and breaking edges by smoothing the image both in spatial and frequency domains. Because of viewpoint invariance, a second order derivative gradient is used instead of a first order derivative. This property resembles the human cognitive process and detects changes in structure in any direction. The edge-based approach is efficient in distinguishing between informative and non-informative frames based on the property of different texture structures. Non-informative frames are smooth, blurred with no clear edges as compared to informative frames as mentioned in Figure 5. Thus, multi-fractal technique analyzes mucosa texture at various scales with local densities *α* ∈ *R* that provides rich descriptors {*E_α_*: *α* ∈ *R*} to categorize different object structures. *E_α_* is the vector of fractal dimensions used for classification.

**Figure 7.**
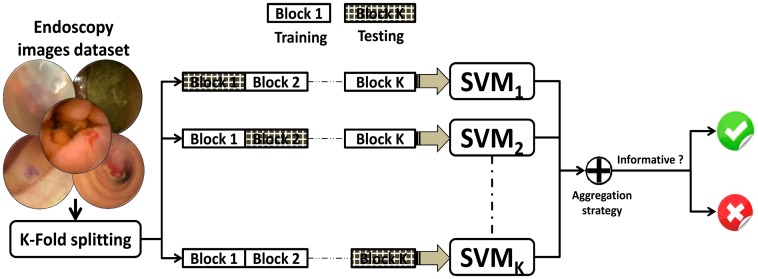
A general framework for training of ensemble SVM (E-SVM).

Based on multi-fractal texture features, we want to classify frames into two categories: informative and non-informative frames. However, in applications like capsule endoscopy, the amount of data in video is extremely diverse and it is impractical to train a single classifier with this range of diverse data. This heterogeneous nature of WCE data makes the performance of a classifier, such as support vector machine (SVM) very limited. This leads to a concept of an ensemble SVM (E-SVM) that showed improved performance over conventional SVM [73–75]. In [76–78], it was experimentally proved that E-SVM is more robust in case of datasets having large variation. It combines the decision of several classifiers to draw the final decision resemble to the approach of consulting several physicians' opinions before reaching to the final decision. Thus, we incorporate the ensemble-based approach for classification presented by Jaffar *et al.* [79]. This ensemble classifier consists of a number of binary SVM classifiers (SVM_1_, SVM_2_,_…_,SVM_K_). Each individual SVM is trained independently on WCE images dataset via a K-fold splitting process as shown in Figure 7. In K-fold splitting, the underlying dataset is split into k non-overlapping folds. Each fold iteratively serves once to estimate the classifier's performance (testing), while the remaining K−1 folds are used to train combination of classifiers. K-fold splitting helps to select those parameters that achieve the best performance on the validation data automatically. Finally, the independently trained several SVMs are aggregated using linear methods such as sum, product and median rule.

In E-SVM, each SVM works on two levels: first using kernel function to map feature vectors into a high-dimensional space and then it separates them into two classes using a hyper decision surface [80]. Usually, three types of kernel function are used such as radial basis function (RBF), linear function, and sigmoid. In the context of classification algorithm, a kernel function is a kind of similarity measure between the input objects. There is no specific criterion for kernel selection because each of them has some degree of variability in performance in different fields. The trade-off between performance and computational complexity is also important to be considered while selecting the kernel function. The SVM algorithm usually depends on efficient optimization of the kernel function parameters. Optimizing kernel parameters is an important step for various tasks such as finding the right shape of the kernel, feature selection, finding the right trade-off between error and margin, outlier detection. We have optimized SVM kernel parameters by gradient descent approach as discussed by Chapelle *et al.* in Chapter 4 [81].The comparative analysis of the classification performance of different SVM kernels using various aggregation schemes is shown in Table 1. Through experiments, it was observed that RBF outperforms other kernel function on the underlying endoscopic dataset. This performance gain comes at the cost of computational complexity. However, the classification task is offloaded to cloud, where the computational complexity is not a crucial factor due to the availability of large computational resources. In aggregation methods, the median rule showed better performance over sum and product rule.

### 3.3. Cloud Service

In remote patient monitoring, clinicians require access to precise and complete information to perform diagnosis correctly. Cloud storage provides ubiquitous access to patients' data in a secure manner with no restrictions on mobility. With a massive increase in data, traditional cloud storage suffers from challenges such as information mining and retrieval. The problem in patient monitoring is not lack of data, but lack of precise and useful information. For instance, in WCE, only a fraction of video frames useful for diagnosis. We have incorporated data summarization in cloud storage in order to process the large amount of WCE data and transform it into precise and useful information. This will solve the problem of on demand access to the required data without browsing enormous redundant data to search for diagnostic related information. There is a variety of cloud vendors (e.g., Amazon, Microsoft, and Google). We selected the Google App Engine [16] because it possesses certain unique features. It is compatible with JavaScript, which is suitable for Android applications. Google App Engine provides high-level security by encrypting its services using secure socket layer. It offers excellent security features by providing an option to set up the access-control restrictions based on the roles assigned to each user. Thus, Google App Engine ensures the security and confidentiality of the patient information owing unique security features. Furthermore, for prototyping, it provides free services. Computationally expensive computer vision tasks (feature extraction and classification) are offloaded to utilize the resources provided by Google App Engine. The summarization and data sharing services are exposed through VMs. The cloud with summarized data can efficiently connect many hospitals and remote patients to provide medical information. It will introduce new methods of accessing patient's information for diagnostic as well educational perspectives. This reduces the problems of reaching medical resources to remote areas because of geographical, resource and time constraints.

## 4. Experimental Evaluation

In this study we evaluated the effectiveness of the proposed framework in terms of: (1) computational time and energy consumption; (2) impact on cloud storage and information retrieval; and (3) video summarization performance. The parameters adopted in these experiments are listed in Table 2. A prototype of the proposed video summarization is developed in the laboratory. The prototype is based on a *local computation* approach, *full offloading*, and *adaptive-offloading*. We used the Samsung Galaxy S4 smartphone running on Android OS 4.2.2 with a Java MIDP emulator and Bluetooth Smart (earlier called Bluetooth low-energy). We used fifteen videos in our experimental analysis. In these fifteen videos, ten were downloaded from open database *Gastrolab* [82] and other five were downloaded from *WCE Video Atlas* [83]. These are the two WCE standard datasets for GI analysis. The collected WCE videos consist of both normal and abnormal images with a rate of two frames per second. For experimentation, these videos are stored in IRU. Smartphone communicates via Bluetooth to IRU. In addition, the smartphone collaborates with the cloud server, which in our case is the Google App Engine. Google App Engine offers multiple APIs including Java, Java 7 runtime with cloud datastore to provide a fully managed schemaless database for storing WCE data [16].

Here, the evaluation is performed on the proposed video summarization framework using three implemented prototypes: *local computation* (smartphone), *full-offloading*, and *adaptive-offloading*. In the first scenario, *local computation*, it is assumed that all the computations were performed on the mobile device. We executed a full video summarization algorithm on the smartphone to measure the summarization performance and energy consumed by the proposed method. Besides, transmitting keyframes to the cloud, no other task was offloaded. In the second scenario, *full-offloading*, complete video was transmitted to Google App Engine for video summarizing and ubiquitous sharing. In the third scenario, the proposed MCATS framework is evaluated by varying the offloading parameters. Here, the application is adaptively offloaded; a set of non-remote functions (redundancy elimination) were executed on a smartphone client and the set of remote functions (multi-fractal texture feature extraction and classification) were offloaded to the Google App Engine.

### 4.1. Evaluation of Computational Time and Energy Consumption

This section focuses on the effectiveness of the proposed framework in minimizing the overall computational time and energy consumption.

#### 4.1.1. Computational Time Estimation Model

Consider the summarization kernel function *Fs* (that includes redundancy elimination and frame classification). Its total execution time *P^L^* on the local server is the sum of the video summary computational time and the generated keyframes transmission time to Google App Engine as mentioned in Equation (11). The execution time *P^F^* for *full-offloading* is shown in Equation (12). In this case, we calculate the total transmission time required for uploading the complete WCE video to the cloud (Google App Engine) plus the time spent by cloud in generating the video summary. Equation (13) shows the total time *P^A^* required by the proposed framework to adaptively generate summaries. This is the time taken by the smartphone to remove redundant frames from video and transmit the processed (short segments) video to the cloud for further processing. This is an adaptive process that depends on the bandwidth, summarization level, and availability of smartphone resources. The proposed framework will be beneficial in terms of time complexity if it satisfies the inequalities mentioned in Equation (14):

(11) $$P^{L}(Fs)=T_{F}^{L}+\frac{V_{K}}{BW}+K$$

(12) $$P^{F}(Fs)=T_{F}^{C}+\frac{V_{F}}{BW}+K$$

(13) $$P^{A}(Fs)=T_{P}^{C}+\frac{V_{S}}{BW}+K$$

(14) $$P^{A}(Fs)<P^{L}(Fs)\&P^{A}(Fs)<P^{F}(Fs)$$

**Figure 8.**
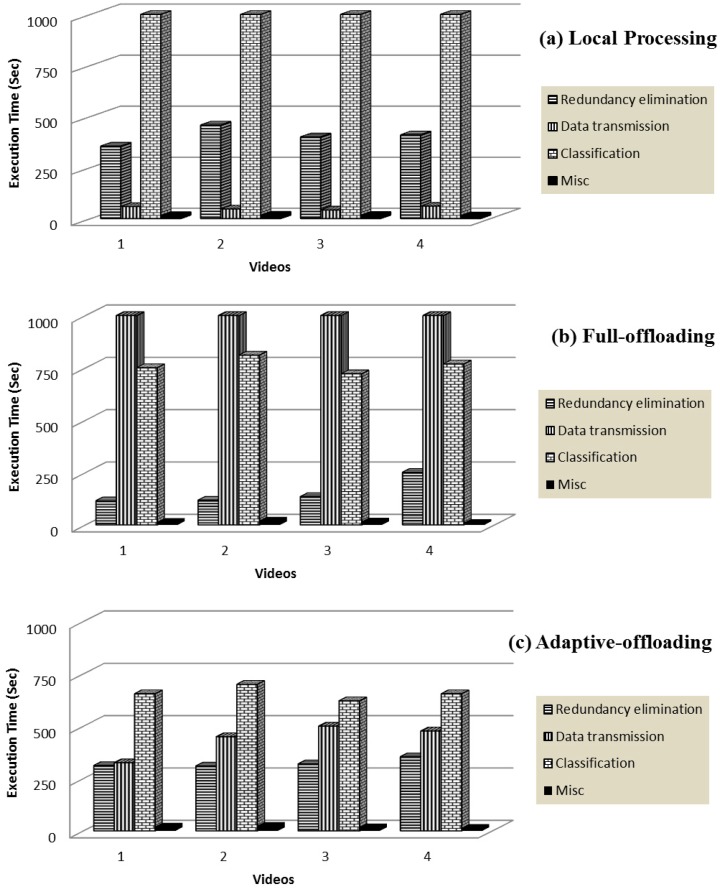
Graph depicts three different computational schemes for WCE video summarization. Each computational scheme consists of three main modules: redundancy elimination, classification, and data transmission. (**a**) *Local processing* scheme took minimum transmission time but maximum redundancy removal and classification time; (**b**) In *full-offloading* approach, data transmission time is significantly higher which make it expensive; (**c**) *Adaptive-offloading* shows a better balance between computational and communication cost which makes it a feasible option for remote data management in long duration.

Figure 8 shows the execution time for different computational strategies dealing with four different WCE videos. In this experiment, a limited bandwidth of 500 kbps is used. Computational time for redundancy removal is significant in both cases: the smartphone operating alone and *adaptive-offloading*. However, in case of *full-offloading*, no processing is required to remove the redundancy because it aims to transmit the complete video to the cloud without processing on smartphone. On the other hand, package transmission time is very high in the *full-offloading* approach as compared to other two approaches. This package transmission time has been reduced substantially in the *adaptive-offloading* approach by locally removing redundant frames and transmitting only the processed non-redundant video segments to the cloud.

Figure 8c shows the effect of the proposed framework that transfers only the non-redundant video frames and offloads the computationally expensive tasks. The smartphone consumed more time on processing and transmission, whereas *adaptive-offloading* took less time. Key-frame execution time reduces significantly on the cloud because the cloud has more computational resources as compared to smartphone. Thus, it can be concluded that the proposed framework outperforms the other two schemes in terms of total execution time.

#### 4.1.2. Energy Consumption Estimation Model

Energy consumption is defined as the total power consumed by the processor from the start to the termination of the application. In local computation, the total energy consumed *E^L^* is calculated in Equation (15). This is a sum of the energy consumed during keyframe generation, and the energy consumed for their transmission to the cloud. Equation (16) shows the energy consumed during the *full-offloading* approach. This is a sum of the energy required for transmitting the original video to the cloud and the energy consumed by the Google App Engine in generating video summaries. Equation (17) describes the formula to calculate the proposed framework's (*adaptive-offloading*) energy consumption. Equation (18) indicates that the proposed framework will be successful in minimizing energy consumption if the mentioned inequalities hold true.

We measure the energy consumption on the smartphone using an energy profiling tool [84]. The functionality of this energy profiling tool is based on three steps: the *Mobile System Monitor*, the *Energy Monitor*, and the *Energy Analyzer*. The *Mobile System Monitor* is responsible for data collection. It monitors mobile's system activity by periodically recording information of the program counter (PC) and process identifier (PID) of the currently executing process. With help of a digital multimeter, the *Energy Monitor* measures the *electric current* being used by the mobile device. According to the energy profiling tool, the voltage variation is extremely small in case of mobile devices; therefore the electric current samples alone are sufficient to determine the energy usage of the system. The Energy Analyzer associates the electric current sample to a PID collected from the Energy Monitor and the Mobile System Monitor respectively. The total energy consumption of a particular process is computed by multiplying the total electric current consumed with time interval of that process:

(15) $$E^{L}(Fs)=\left(T_{F}^{L}*EC^{C}\right)+\left(\frac{V_{K}}{BW}+K\right)*EC^{T}$$

(16) $$E^{F}(Fs)=\left(T_{F}^{C}*EC^{I}\right)+\left(\frac{V_{F}}{BW}+K\right)*EC^{T}$$

(17) $$E^{A}(Fs)=\left(T_{F}^{L}*EC^{C}\right)+\left(\frac{V_{S}}{BW}+K\right)*EC^{T}$$

(18) $$E^{A}(Fs)<E^{L}(Fs)\&E^{A}(Fs)<E^{F}(Fs)$$

**Figure 9.**
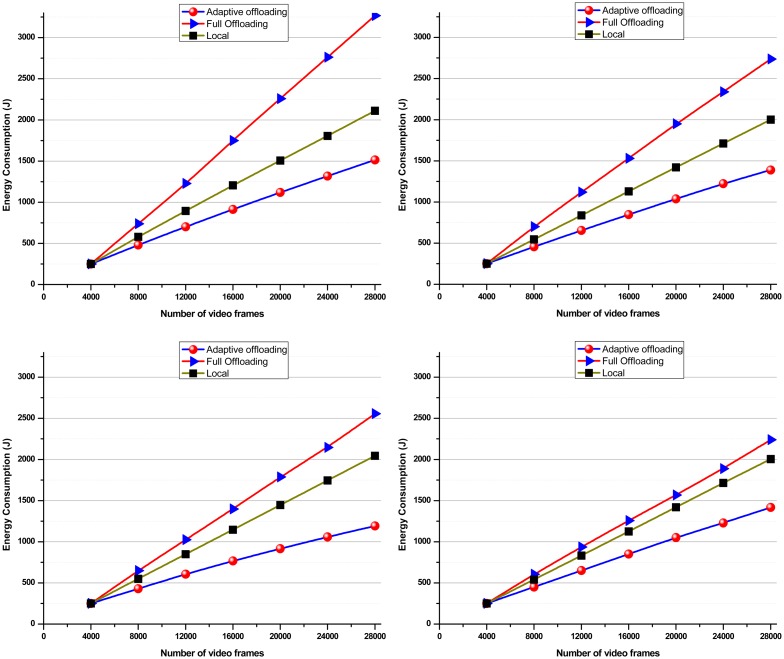
Graph describing in detail the effect of three computational scenarios: (1) *local processing*; (2) *full-offloading*; and (3) *adaptive-offloading* method on energy consumption in generating video summaries.

Figure 9 shows the total energy consumed during execution of video summary on four WCE videos. This provides strong evidence that for long durations and computationally intensive tasks such as video summarization, local execution severely affects the smartphones' energy. Although it mitigates the data transmission cost (because only key-frames are transmitted), it drains the smartphone resources (battery and enrgy); this is not acceptable from the users' point of view. Execution traces of video summaries for *full-offloading* scenario provide different results. This can be explained by the fact that *full-offloading* needs more transmission power because it has to push the complete video to the cloud. Consequently, the transmission load increases. Furthermore, the energy consumption of transmitting a fixed amount of data is inversely proportional to the available bandwidth [85]. Thus, in dealing with large diagnostic data over frequently fluctuating bandwidth conditions, full off-loading fails to save smartphones' energy. In such scenarios, performing computations on local servers (smartphones) might be a better choice. However, smartphones are constrained in terms of computation power and storage. Thus, it is not feasible to utilize the smartphone's resources beyond a limit; this will affect the user experience. The proposed framework partitions the computation tasks between local and cloud servers to reduce energy consumption. It is a carefully designed scheme that efficiently manages available resources by weighing the benefits of transmission and computation costs as illustrated in Figure 9.

It can be inferred from Figure 9 that the energy consumed by the proposed method is significantly low as compared to other mentioned techniques. In this experiment, we varied video size and kept all other parameters (bandwidth, smartphone resources and threshold *τ*) fixed. The graph given in the Figure 9 illustrates the variational impact of video size on total energy consumption. From graph, one can see that the total energy consumption in three computational scenarios grows almost linearly as the duration of the input video gets increase.

Table 3 shows the approximate energy consumption both at smartphone and cloud server of WCE video summarization for three different computational scenarios. The WCE video consists of one hour at frame-rate: two frames per second. The *full-offloading* approach consumes more energy than *local* and *adaptive-offloading* approaches because of transmission load. *Local processing* approach minimizes the transmission load at the cost of conducting computational intensive task locally, *i.e.*, classification. However, the execution of such energy-hungry task at smartphone is not a feasible option. Cloud provides an ideal environment for such resource-hungry task than smartphones and this is very clear from the computation mentioned in the Table 3. According to the statistics computed in Table 3, the *adaptive-offloading* consumes 34% and 19% less energy than *full-offloading* and *local* approaches. In addition, it decreases the smartphone's energy consumption by 60% compare to *local processing* approach. The Samsung Galaxy S4 has a 2600 mAh battery (9.8 Wh), approximately equal to 35,568 Joules (J). Most of the wireless capsule batteries have life span of about eight hours [4], typically enough time for capsule to image the entire small bowel as it passes through. Although most capsules are naturally expelled within 72 h of ingestion, only the first eight hours are significant for capturing the visual frames of the gastrointestinal because after eight hours wireless capsules are unable to capture images. Therefore, the energy estimated above is sufficient to monitor the WCE procedure for 8 h and as well as to run the native applications of the smartphones. Therefore, *adaptive-offloading* is the most suitable approach to run in a resource constrained environment such as smartphone.

### 4.2. Impact on Cloud Storage and Information Retrieval

To evaluate the effect on storage cost, the proposed summarization scheme along with other two state-of-the-art techniques were compared with a traditional baseline solution (BS). The BS schemes usually stores the entire video data instead of summarized one. The other two state-of-the-art techniques are: (1) our recent visual attention driven summarization method [21]; and (2) a domain specific endoscopy summarization scheme presented by Iakovidis *et al.* [22]. In [21], we presented a summarization technique based on visual attention that generates summary of the hysteroscopy video. The visual attention value of each frame in the video was calculated using inter-frame motion, color changes, and texture-based segmentation. Iakovidis *et al.* proposed an endoscopy video summarization technique that uses an unsupervised image mining method for summary generation. In this scheme, non-negative matrix factorization [86] is used to extract a set of orthogonal vectors. Then, on the basis of these orthogonal vectors, video frames are clustered to remove redundant frames. The number of frames extracted is controlled by a parameter that can be adjusted according to the level of detail required. In this set of experiments, WCE videos summaries were generated using three different video summarization methods with varying threshold *τ*. The value of *τ* is normalized in the range [0, 1] that determines the level of summarization. Threshold *τ* carrying small value generates a summary consist of more detail, *i.e.*, summarization level is low. For high-level summarization, a large value is chosen for *τ* to extract only the most important frames.

**Figure 10.**
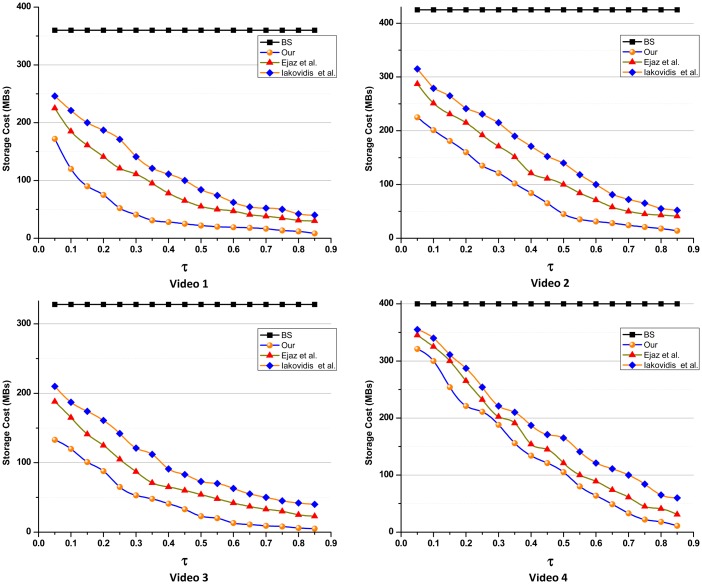
The impact of level of summarization on storage cost.

Figure 10 shows that how threshold *τ* improve the efficient utilization of the storage. It can be seen that for all three summarization schemes, increase in *τ* from zero to one significantly decrease the storage costs for the summarized videos as compared to the traditional storage solutions. For the proposed summarization method, when *τ* is greater than 0.2, the storage requirement is reduced to 50% as compared to the baseline approach. Moreover, this reduction in data is beneficial for efficient image browsing. In addition, the resource effectiveness nature of the proposed summarization minimizes bandwidth consumption. Moving data of terabytes in inter (from the clients to the cloud) and intra (within the cloud model) cloud can be very expensive and time consuming. Existing cloud storage gateways leverage wireless area network optimization and deduplication schemes [87]. These duplication and compression methodologies reduce the amount of data traffic to the cloud by 10%–30%. Furthermore, for threshold *τ* = 0.5, the storage cost of the underlying four videos approximately reduces to 72%, 45%, and 41% while using the proposed, Ejaz *et al.* and Iakovidis *et al.* schemes for summarization respectively. This reduction in the non-informative and redundant data reduces the data traffic.

### 4.3. Subjective Evaluation of the Proposed Video Summarization

The proposed summarization performance is based on subjective rating done by experts. A group of five gastroenterologists was requested to select keyframes from given WCE videos. These gastroenterologists have an average experience of ten years in the medical field. The keyframes were selected based on a diagnostic point of view (having clear view of mucosal). The keyframes manually selected were used as the ground truth for comparison with keyframes extracted by the automated methods. Comparison between manually generated summaries (keyframes) and automated generated summaries is performed using standard metrics: Recall, Precision and F-measure. These three metrics are defined as:

(19) Recall=TPTP+FN

(20) $$\mathrm{Pr}\text{ecision}=\frac{TP}{TP+FP}$$

(21) F−Measure=2*Recall*PrecisionRecall+Precision

where *TP* is true positive, *FP* is false positive, and *FN* is false negative. A frame is true positive, if it is selected by both a human user and the automatic technique. A frame selected only by the technique and not by a human user is false positive. False negative is the frame that is selected by a user but not by the technique. In the current scenario, *Recall* is the probability that an informative collection of WCE frames is generated by the technique while, *Precision* is the probability that generated summary is informative. These two measures: *Recall* and *Precision* have a trade-off. To eliminate this trade-off, *F-Measure* is used. *F-Measure* is the weighted harmonic mean of *Recall* and *Precision* that provides a better understanding of the generated summaries [88]. A comparative analysis of summaries generated by the proposed method, Ejaz *et al.* [21] and Iakovidis *et al.* [22] were conducted using *Recall, Precision* and *F-Measure* metric. Table 4 illustrates that the proposed method achieves high values for *Recall*, *Precision* and *F-Measure*. High *Recall* values demonstrate the ability of a particular technique to detect more informative frames as compared to other techniques while the high *Precision* values indicate the level of preciseness in the selected summarized frames. Moreover, the average *F-measure* of the proposed technique for fifteen videos is 0.82 that outperforms the other techniques. This indicates that the generated summaries are more informative, precise, and are closer to manually selected summaries, *i.e.*, ground truth.

**Figure 11.**
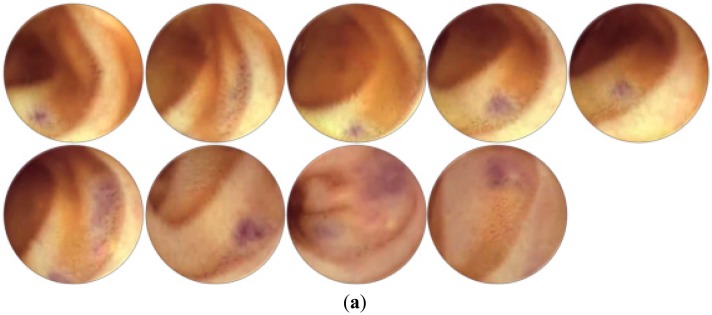

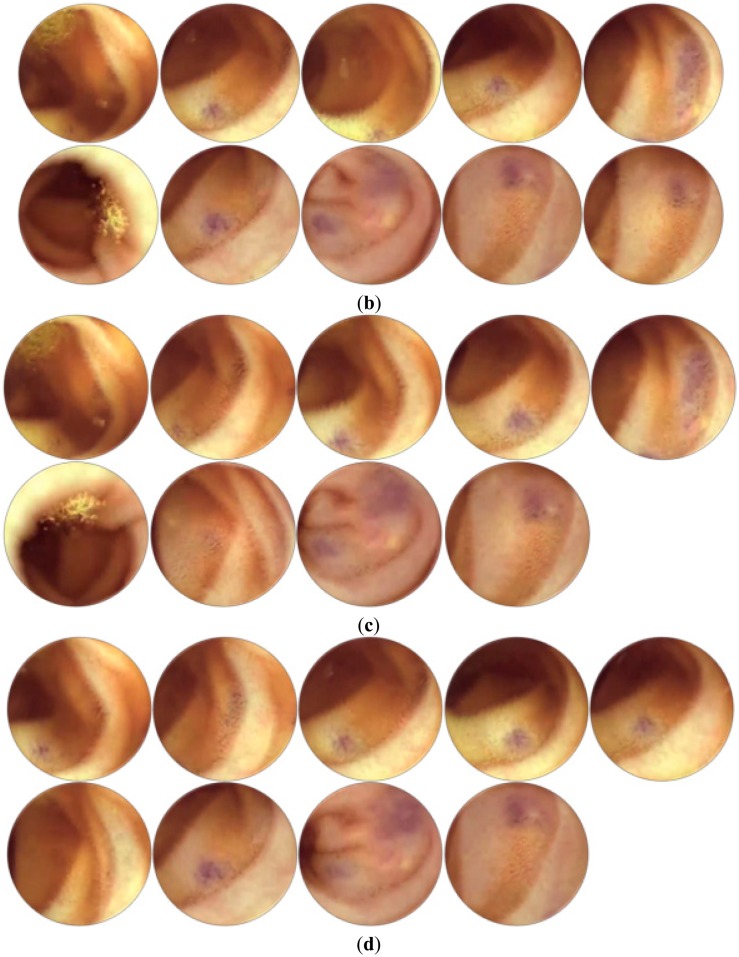
Comparison of manual summary with summaries generated by three different summarization techniques. (**a**) Ground truth; (**b**) Summaries generated by the Iakovidis *et al.* [22]; (**c**) Summaries generated by the Ejaz *et al.* method [21]; (**d**) Summary generated by the proposed method.

Figure 11a, shows those frames of the WCE video that were selected manually as a ground truth. The underlying video-shot is consists of 340 frames. The video-shot was captured by SYNMED [89] for the purpose to diagnose an abnormality of the gastrointestinal called phlebectasia. This video was downloaded from YouTube [90]. Few frames are informative and almost 80% frames are redundant. There are some frames that are usually selected as informative frames by summarization technique due to bubble patterns. The bubble pattern creates a deceptive vision of tissues being obstructed. Figure 11b–d shows summaries of this video generated by the Iakovidis *et al.*, Ejaz *et al.* and the proposed techniques respectively.

The Iakovidis *et al.* and Ejaz *et al.* techniques work efficiently in removing the redundant frames, however they fail to detect and discard the non-informative frames (frames with bubble patterns) as depicted in the first column of Figure 11b,c. These schemes efficiently eliminate redundancy in video frames but fail to differentiate between non-redundant and informative frames. Figure 11d shows that the proposed method outperforms other two techniques by removing the redundant as well as the non-informative frames.

## 5. Conclusions

In this study, we formulated the problem of an energy-efficient wireless capsule's data management and proposed a mobile-cloud assisted video summarization framework its solution. This is a two-fold solution that deploys the capabilities of mobile-cloud computing and image processing techniques. The large sensor data captured by wireless capsule sensor is summarized to remove redundant and non-informative frames. The summarization process is based on two steps: (1) elimination of redundant video frames and (2) classification of non-redundant frames into informative and non-informative. For redundancy elimination, net similarity between two WCE video frames is calculated by linearly combining the two similarity measures: Jeffrey-divergence and Boolean series-based correlation. Then, rotational and translation invariant multi-fractal texture features are extracted to classify each frame as informative or non-informative. Due to the complex and diverse nature of WCE visual contents, an ensemble-based classification is performed using support vector machine. The proposed redundancy elimination is a light weight process but feature extraction and classification are computationally intensive tasks. Therefore an adaptive approach is employed to partition the processing tasks between smartphone and cloud servers. The light-weight redundancy elimination step is performed at patients' smartphone, whereas, classification task is offloaded to cloud.

The simulation results indicate that the proposed summarization scheme efficiently extracts semantically important frames from videos; as a result, the overall size of video data is reduced. This reduction in data size reduces network transmission cost, storage space, and most importantly the browsing time. Furthermore, the adaptive offloading mechanism efficiently partitions the processing tasks between smartphone and cloud by considering the communication and computational trade-off. The simulation result recommends that the proposed framework would provide medical specialists fast and easy access to vital information anytime/anywhere during WCE procedure.

Our approach uses an adaptive threshold that reduces smartphone's computational and communication burden and only transmits limited number of frames to cloud. However, it is not trivial to determine efficient value of threshold, since the decision between redundant and non-redundant tends to be subjective. In future, we have intention to formulate current wireless capsule's data management problem as a multiple-objective optimization driven summarization framework. In which WCE data can be efficiently managed by solving the multi-objective optimization problem based on the frame importance. Different summarization objectives such as *minimum summary length* and *maximum information coverage* can be accomplished according to the requirements of gastroenterologists.

## Figures and Tables

**Table 1.** The comparative analysis of the classification performance of different SVM kernels using various aggregation schemes.

**SVM Kernel**	**Aggregation Strategy**		

	**Product Rule Accuracy %**	**Sum Rule Accuracy %**	**Median Rule Accuracy %**
**RBF**	76.55	78.21	**79.15**
**Linear**	62.21	65.12	**66.51**
**Sigmoid**	40.12	43.25	**44.51**

**Table 2.** Parameters of the simulation testbed.

**Parameter**	**Description**
*Fs*	Summarization kernel function
*V_K_*	Keyframes extracted from video
*V_F_*	Complete WCE video
*V_S_*	Short video segments of original WCE video after removing redundancy.
*P^L^*	Sum of time required for keyframe extraction on smartphone and their transmission to the cloud.
*P^F^*	Total time required to transmit original video to the cloud and keyframe extraction.
*P^A^*	Time required by the proposed method for video summarization
$T_{F}^{L}$	Complete video Summary execution time on local server (smartphone).
$T_{F}^{C}$	Complete video Summary execution time on the cloud.
$T_{P}^{C}$	Time required classifying processed video frames into informative and non-informative.
*E^L^*	Energy required for executing the complete video summarization on smartphone.
*E^F^*	Energy required for transmitting complete video to the cloud.
*E^A^*	Energy required for video redundancy removal and frame classification using proposed method.
*EC^C^*	Power required for computation of summarization algorithm in one second on smartphone.
*EC^T^*	Power required for transmitting data via Wi-Fi in one second on smartphone.
*EC^I^*	Smartphone' power used in one second in an idle state.
*BW*	Available bandwidth.
*K*	Miscellaneous: time required for receiving data from WCE Image recorder, monitoring mobile resources *etc.*

**Table 3.** Approximate energy consumption (in Joule) both at smartphone and cloud server of WCE video summarization for three computational scenarios: (1) *local computation*; (2) *full-offloading* and (3) *adaptive-offloading* computation method.

**Computational Scenario**		**Local**	**Full-Offloading**	**Adaptive-Offloading**
**Energy consumption at smartphone**	Bluetooth low-energy	67	62	75
	Reduction of redundancy	405		388
	Classification	1689		
	data transmission	89	1352	389
**Energy consumption at Cloud**	Reduction of redundancy		250	
	Classification		1090	852
**Smartphone's total energy (J)**		**2250**	**1414**	**852**
**Total energy (J)**		**2250**	**2754**	**1844**

**Table 4.** *Recall* (R), *Precision* (P), and *F-Measure* (F) scores for different techniques.

	**Iakovidis *et al.***			**Ejaz *et al.***			**Proposed**		
		
	**R**	**P**	**F**	**R**	**P**	**F**	**R**	**P**	**F**
**1**	0.62	0.71	0.66	0.71	0.70	0.70	0.78	0.84	0.81
**2**	0.71	0.76	0.73	0.72	0.75	0.73	0.80	0.82	0.81
**3**	0.69	0.77	0.73	0.68	0.78	0.73	0.83	0.91	0.87
**4**	0.81	0.68	0.74	0.85	0.88	0.86	0.79	0.84	0.81
**5**	0.67	0.69	0.68	0.74	0.78	0.76	0.80	0.87	0.83
**6**	0.72	0.71	0.71	0.72	0.75	0.73	0.82	0.88	0.85
**7**	0.75	0.70	0.72	0.75	0.77	0.76	0.81	0.84	0.82
**8**	0.70	0.72	0.71	0.71	0.74	0.72	0.79	0.78	0.78
**9**	0.66	0.67	0.66	0.69	0.67	0.68	0.83	0.85	0.84
**10**	0.65	0.68	0.66	0.67	0.75	0.71	0.85	0.88	0.86
**11**	0.73	0.71	0.72	0.72	0.74	0.73	0.79	0.83	0.81
**12**	0.76	0.77	0.76	0.75	0.71	0.73	0.78	0.72	0.75
**13**	0.74	0.75	0.74	0.73	0.75	0.74	0.83	0.87	0.85
**14**	0.69	0.72	0.70	0.79	0.82	0.80	0.80	0.86	0.83
**15**	0.67	0.70	0.68	0.78	0.74	0.76	0.79	0.82	0.80
**Average**	**0.70**	**0.72**	**0.71**	**0.73**	**0.76**	**0.74**	**0.81**	**0.84**	**0.82**
